# Electrocardiogram: the saviour for this patient

**DOI:** 10.1007/s12471-015-0793-3

**Published:** 2015-12-08

**Authors:** A. Chawla, V. Gaikwad, S. Swarup

**Affiliations:** 1 Department of Diagnostic Radiology, Khoo Teck Puat Hospital, 90 Yishun Central, 768828 Yishun, Singapore; 2 Department of Accident and Emergency, Khoo Teck Puat Hospital, Yishun, Singapore

**Fig. 1:**
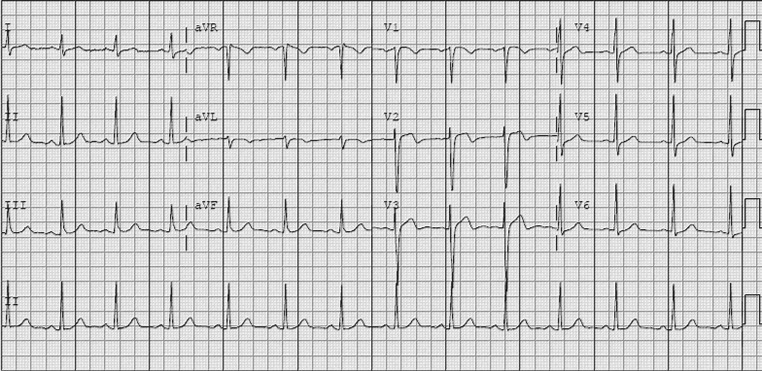
Electrocardiogram on admission

A 42-year-old man presented to the emergency department with a history of central non-radiating chest pain lasting for 25 min, 2 h back. The pain was associated with vomiting and sweating, and subsided spontaneously. He was pain free at the time of presentation. He had had similar episode 3 weeks previously, which lasted less than a minute while he was rock climbing. There was history of hyperlipidaemia for which he was not taking any medication. His physical examination including cardiac evaluation was within normal limits. The cardiac enzymes were also normal. His electrocardiogram on admission is shown in Fig. 1. What would your diagnosis be and what would you expect to find on cardiac catheterisation?

Answer

You will find the answer elsewhere in this issue.

